# Differences in health literacy profiles of patients admitted to a public and a private hospital in Melbourne, Australia

**DOI:** 10.1186/s12913-018-2921-4

**Published:** 2018-02-22

**Authors:** Rebecca L. Jessup, Richard H. Osborne, Alison Beauchamp, Allison Bourne, Rachelle Buchbinder

**Affiliations:** 1 Health Systems Improvement Unit, Centre of Population Health Research, School of Health and Social Development, Deakin University, Geelong, Australia; 2 Monash Department of Clinical Epidemiology, Cabrini Institute, Melbourne, Australia; 3 Department of Epidemiology and Preventive Medicine, School of Public Health and Preventive Medicine, Monash University, Melbourne, Australia

**Keywords:** Health literacy, Literacy, Equity, Hospitals, Quality, Safety

## Abstract

**Background:**

Health literacy refers to an individual’s ability to find, understand and use health information in order to promote and maintain health. An individual’s health literacy may also be influenced by the way health care organisations deliver care. The aim of this study was to investigate the influence of hospital service type (public versus private) on individual health literacy.

**Methods:**

Two cross-sectional surveys were conducted using the Health Literacy Questionnaire (HLQ), a multi-dimensional self-report instrument covering nine health literacy domains*.* Recently discharged private patients (*n* = 3121) were sent the survey in English, public patients (*n* = 384) were sent the survey in English, Arabic, Chinese, Vietnamese, Italian or Greek. Eligibility included hospitalisation ≥24 h in last 30 days, aged ≥18 years, no cognitive impairment. Odds ratios were used to assess differences between hospital sociodemographic and health related variables. ANOVA and Cohen’s effect sizes compared HLQ scores between hospitals. Chi square and multiple logistic regression were used to determine whether differences between private and public hospital HLQ scores was independent of hospital population sociodemographic differences. ANOVA was used to review associations between HLQ scores and subgroups of demographic, health behaviour and health conditions and these were then compared across the two hospital populations.

**Results:**

Public hospital participants scored lower than private hospital participants on eight of the nine health literacy domains of the HLQ (scores for *Active Appraisal* did not differ between the two samples). Six domains, five of which in part measure the impact of how care is delivered on health literacy, remained lower among public hospital participants after controlling for age, education, language and income. Across both hospital populations, participants who were smokers, those who had low physical activity, those with depression and/or anxiety and those with 3 or more chronic conditions reported lower scores on some HLQ domains.

**Conclusions:**

Our finding of lower health literacy among patients who had received care at a public hospital in comparison to a private hospital, even after adjustment for sociodemographic and language differences, suggests that private hospitals may possess organisational attributes (environment, structure, values, practices and/or workforce competencies) that result in improved health literacy responsiveness.

**Electronic supplementary material:**

The online version of this article (10.1186/s12913-018-2921-4) contains supplementary material, which is available to authorized users.

## Background

Health literacy refers to an individual’s ability to find, understand and use health information in order to promote and maintain health [1]. Having a sufficient level of health literacy is required for patients to adequately interact and ask questions of their health professionals, manage medications and appointments, actively monitor their conditions, adjust regimens in response to changes in disease progression, and to implement and maintain lifestyle changes in order to manage and improve health.

Many studies have examined the association between health-related reading and comprehension skills (*functional* health literacy) and health and hospital outcomes. These studies have found that compared to individuals with higher health literacy, those with lower health literacy have poorer knowledge about their chronic disease [2–5], lower capacity for self-management and medication adherence [6, 7], poorer disease outcomes [3, 5, 8], increased risk of hospitalisation [9, 10], and increased mortality [11]. However, these studies have focused on health literacy as an individual ability, leading to a formative conceptualisation of health literacy as a patient ‘problem’ that health professionals have to overcome [12]. This fails to recognise the complex interplay between an organisation’s delivery of health care and an individual’s knowledge and skills. An individual’s health literacy impacts on how they manage interactions and communication with a range of health professionals with different experiences and approaches in their delivery of health advice. Health care professionals who are encouraged to spend time with patients, be supportive and sensitive to differences in cultural and social needs, and adapt health education to the requirements of the individual, will impact on a patient’s ability to understand and manage their condition [13]. In addition, the environment within which health care is delivered (location, signage, and inviting design) and the methods and modes of delivery of health care messages (appointment letters, brochures, and instructional material) all have an impact on how effectively patients can interact with a health care organisation [14].

The term ‘health literacy responsiveness’ has recently been defined as the provision of health services that promote supportive, equitable participation and access in a way that meets the diverse health literacy needs and preferences, and which is achieved through supportive culture and leadership, systems, policies and practices, and an effective workforce [13]. Health care organisations are increasingly being called upon to measure and address the health literacy needs of the populations they serve [15–17]. However no research to date has compared the health literacy of patient populations seeking care from health care organisations with different service delivery models.

Australia has a universal healthcare system that provides healthcare for all citizens. For inpatient care, patients can be treated in public hospitals, where care is provided free of charge and is paid for by the government. Alternatively, individuals can choose to purchase private health insurance and be treated in a public or private hospital. Private hospital care is subsidised by the government to the value of public hospital costs, with additional costs borne by the individual’s private insurer (or paid for by the individual in the absence of private health insurance) [18]. Private hospital patients have the advantage of choosing their physician (patients without private health insurance are assigned a staff physician) and may get priority access to many interventions or treatments, whereas uninsured patients attending public hospitals may be placed in a queue. This dual health care system provides a unique opportunity to not only determine whether or not health literacy of patients differ according to where they seek care (private or public systems), but to also explore the health literacy responsiveness of these two settings. The aim of this study was to use a multidimensional health literacy instrument to measure, compare and contrast the health literacy of a private and a public hospital inpatient population.

## Methods

### Study design, sample and populations

We used data from two separate cross sectional surveys of recently hospitalised patients attending either a private hospital [19] or a public hospital [20], both in Melbourne, Australia. Both surveys were undertaken as part of a program of research to better understand the needs of patients attending these health services, and to identify strategies that the hospitals could adopt to better serve their populations. These hospitals are the sites of employment of two of the investigators (RJ, RB). The first survey was conducted in a 508-bed not-for-profit private hospital located in Melbourne’s south-eastern suburbs. According to data from the Australian Socio-Economic Indexes for Areas (SEIFA), this hospital serves a population where income, education, and employment are higher than Victorian state averages [21]. In contrast, the second survey was conducted in a 400-bed public hospital located in Melbourne’s northern suburbs utilized by individuals with lower income, lower education, greater likelihood of having been born overseas and speaking a language other than English at home, and higher rates of unemployment than Victorian state averages [20].

The survey protocols were similar at both sites and have been described in detail previously [19, 20]. For both surveys, eligibility included hospitalisation for a minimum of 24 h (to exclude day stay patients attending for day medical procedures, day oncology, dialysis or those briefly attending emergency) in the past 30 days, aged 18 or over, no known cognitive impairment, and discharged directly to home. The private hospital survey only included patients who understood written English, however, as the survey can be completed with assistance, patients whose first language was not English could still respond. Due to the high number of migrants located in the public hospital catchment area the survey and accompanying materials (including pre-notification and reminder letters) at this hospital were translated into five of the top 10 most spoken language: Arabic, Chinese, Vietnamese, Greek and Italian. The translation methods have been described in detail previously [20] and are outlined in brief below.

To maximise survey response rate at each site, each participant was sent a pre-notification letter (where applicable for the public hospital, this was in the participant’s first language). A week later, participants were mailed the participant information and consent form, the HLQ and a brief survey of sociodemographic and health-related information in their language. A late return/reminder letter was sent two weeks following the survey. Each of the letters at each phase (pre-notification, letter accompanying the survey, and reminder letter) was personally addressed to the participant, from the Chief Executive Officer of each hospital, and encouraged patients to participate in order to assist the hospital to improve services.

Data collection at the private hospital took place from January to August 2014. As this was the first site of data collection, a pilot test of the mail out process was conducted. In January 2014, 50 randomly selected eligible participants were sent the surveys, followed by 100 in the second month. From March through to August, *all* individuals who met the eligibility requirements and had been hospitalised in the preceding month were mailed surveys, with a total of 9341 surveys sent.

Data collection at the public hospital took place over 6 months from January to June 2015. As this survey was conducted on a smaller budget, 500 participants were recruited per month. All eligible patients who spoke Arabic, Chinese, Vietnamese, Greek or Italian were included, with the remaining patients selected using computer assisted random sampling from eligible English speakers to generate 500 invitations per month. Approximately a third of participants each month were non-English speaking. Envelopes returned with ‘not known at this address’ were replaced with another randomly selected patient from the same month. This resulted in 3252 surveys being sent to public hospital attendees.

This study was approved by the Northern Health, Cabrini Hospital, Deakin University and Monash University Human Research and Ethics Committees.

### Measures

#### Health literacy questionnaire (HLQ)

The HLQ was developed using a validity-driven approach [22]. It has demonstrated strong construct validity and reliability in a wide range of contexts, including within the hospital setting and with patients with chronic and complex conditions [22–26]. It comprises 44 items covering nine independent domains*: 1. Feeling understood and supported by healthcare providers, 2. Having sufficient information to manage my health, 3. Actively managing my health, 4. Social support for health, 5. Appraisal of health information, 6. Ability to actively engage with healthcare providers, 7. Navigating the healthcare system, 8. Ability to find good health information,* and *9. Understanding health information enough to know what to do.* Each domain consists of 4 to 6 items. The items in the first five domains are scored from 1 to 4 (ranging from *strongly disagree* = 1, to *strongly agree* = 4), while the last four domains are scored 1 to 5 (*cannot do* or *always difficult* = 1, to *very easy* = 5). Each domain is calculated by adding up the scores for each item within the domain, divided by the number of items within the respective domain. Each HLQ domain has been demonstrated to be conceptually distinct and measure independent constructs using confirmatory factor analysis in Australia [23, 26, 27] and in other countries [28]. Consequently, a total score is not generated [26].

The HLQ was translated from English into Arabic, Chinese (simplified, so it was suitable for both Cantonese and Mandarin speakers), Vietnamese, Italian and Greek. The translation process consisted of forward and then backward translation, followed by consensus meetings with translators, bilingual speakers and one of the authors (RHO) to ensure that the revealed meaning of items was consistent with what was intended through reference to a detailed item intent document.

#### Interpretation of domain scores

The HLQ was designed to measure both innate ability, as well as an individual’s self-reported experience of interacting with health care organisations. Specifically, scores for *Actively managing my health, Social support for health,* and *Appraisal of health information* reflect the health literacy skills and needs of an individual. In contrast, scores for *Feeling understood and supported by healthcare providers* and *Having sufficient information to manage my health* provide insight into an individual’s interaction with the health system, and may provide guidance around the responsiveness of organisations in addressing individual health literacy needs. Scores for *Ability to actively engage with healthcare providers, Navigating the healthcare system, Ability to find good health information and Understanding health information enough to know what to do* provide insight into both individual and organisational health literacy strengths and weaknesses.

#### Health behaviours and chronic diseases

The following socio-demographic, health behaviour and chronic disease variables were also collected: sex, age, height, and weight (for Body Mass Index), living arrangement, country of birth, educational attainment, employment status, household income, health insurance status, use of the Internet, attendance at any emergency department (ED) in the previous 12 months, participation in physical activity, smoking status, alcohol intake, and presence/absence of chronic diseases. As the HLQ allows for someone to help with the completion of the survey, we also collected details on primary language spoken at home as some non-English speakers from both hospitals were able to respond through the assistance of family in completing the survey. In addition, at each hospital, age, sex and service use information from the hospital’s data warehouse for both respondents and non-respondents was extracted.

### Statistical analysis

We used SPSS® version 22 [29] to estimate the odds of having particular social, economic, health and hospital usage variables if the participant attended the public hospital versus the private hospital. We conducted a one-way between-groups analysis of variance to compare health literacy scores between the two hospital samples.

We then used chi square test and multiple logistic regression (as the data did not meet the assumptions of normality, linearity of relationship and homogeneity of variances) to determine whether the association between hospital of attendance and HLQ domain scores is independent of sociodemographic conditions between the two populations. Variables selected for regression were those previously shown to be independently associated with low health literacy and included age over 65 years, speaking a language other than English, education, and income less than $30,000 [25]. We used backward elimination as the variable selection strategy, with entry level set at *p* = 0.10 and exit at *p* = 0.05. Backward elimination was chosen as, while a strong relationship between health literacy and certain variables such as education and ethnicity has previously been demonstrated using functional health literacy tools, these relationships may differ when a broader multidimensional instrument is used.

Dichotomous variables included in the regression were: age (< 65, ≥65), education (did not complete secondary school, completed secondary school or above), language (English, non-English), income (<$30,000, ≥$30,000). Logistic regression coefficients were transformed to odds ratios (OR), which refer to the odds that attendance at the public hospital (reference variable) is a risk factor for lower HLQ domain scores.

Finally, we performed a one-way between-groups analysis of variance to compare health literacy between subgroups of people within each hospital, according to demographic (male or female, aged ≥65 or < 65) health behaviour (BMI > 25 or ≤25, Alcohol >2glasses/ day or ≤2 glasses/ day, smoker or non-smoker, physical activity ≥2.5 h/week or < 2.5 h/week) and clinical characteristics (presence or absence of depression/anxiety, lung condition, cancer, diabetes mellitus, stroke, heart condition, back pain, arthritis, three or more chronic conditions) within each hospital population. Cohen’s d effect sizes (ES) for standardised difference in means were calculated using Stata® software [30]. The pooled standard deviation (PSD) was used as the denominator, the difference within domains as the numerator, and a *p* < 0.05 was regarded as statistically significant for all tests. ESs describe the magnitude of differences in domain scores across these variables for each hospital. Scores between 0.20–0.50, 0.50–0.80 and greater than 0.80 are considered small, medium and large respectively [31]. Comparing these data across hospitals is useful for investigating whether or not there are commonalities across the two hospitals with respect to associations between HLQ scale scores and demographic and clinical variables.

## Results

Completed surveys were received from 3121 (33%) participants from the private hospital, and 384 (13%) participants from the public hospital (Table 1). The proportion of respondents and non-respondents who were female was similar for the public hospital (49% vs 51%), but slightly less respondents than non-respondents were female at the private hospital (52% vs 59%). At both hospitals, a larger proportion of respondents were older (≥ 65 years) compared with non-respondents (55% vs 40% of non-respondents at the public hospital and 60% vs 48% at the private hospital). Types of admissions were similar between respondents and non-respondents at both hospitals, although a much larger proportion of admissions were planned versus unplanned at the private hospital.

**Table 1:** Characteristics of the respondents and non-respondents of the public and private hospital surveys and comparison of private versus public hospital respondents^a^

	Public hospital		Private hospital		Odds Ratio^a^ (95% CI)
	Respondents (*n* = 384) *n* (%)	Non-respondents (*n* = 2616) *n* (%)	Respondents (*n* = 3121) *n* (%)	Non-respondents (*n* = 6055) *n* (%)	
Female	188 (49%)	1334 (51%)	1621 (52%)	3577 (59%)	0.88 (0.72, 1.10)
Age ≥ 65 years	211 (55%)	1040 (40%)	1875 (60%)	2898 (48%)	0.86 (0.69, 1.06)
Admission type					
Planned admission	27 (7%)	175 (7%)	1756 (57%)	3003 (50%)	^b^
Emergency Department	350 (91%)	2267 (87%)	905 (29%)	2026 (34%)	^b^
Maternity	7 (2%)	189 (7%)	218 (7%)	588 (10%)	^b^
Unscheduled community	n/a	n/a	230 (7.4)	438 (7%)	^b^
Lives alone	70 (23%)		635 (20%)		0.88 (0.67, 1.16)
Not completed secondary education	246 (67%)		1041 (33%)		3.90 (3.10, 4.90)
English spoken at home	290 (76%)		2992 (96%)		9.09 (7.14, 12.5)
Currently employed	98 (26%)		988 (32%)		0.74 (0.58, 0.95)
Household income <$30,000	149 (39%)		413 (13%)		4.15 (3.30, 5.23)
Receiving government benefits (income $$30,000)	234 (64%)		1333 (43%)		2.09 (1.68, 2.60)
Body Mass Index ≥25 (overweight)	252 (78%)		1591 (58%)		2.59 (1.97, 3.40)
Body Mass Index ≥30 (obese)	130 (40%)		566 (20%)		2.63 (2.07, 3.34)
Smoker	29 (8%)		98 (3%)		2.75 (1.81, 4.18)
Drinks alcohol daily	43 (12%)		930 (30%)		0.30 (0.22, 0.42)
Drinks more than 2 glasses alcohol daily	16 (4%)		214 (7%)		0.60 (0.36, 1.01)
Uses internet < once a month	164 (43%)		635 (20%)		2.73 (2.17, 3.44)
Private Health Insurance	111 (31%)		2918 (95%)		39.12 (29.95, 51.09)
Attendance at ED in last 12 months	341 (91%)		1653 (53%)		8.98 (6.24, 12.93)
Help with completion of survey	151 (40%)		821 (26%)		2.76 (2.07, 3.69)
≥ 2.5 h of physical activity/ week	211 (55%)		2230 (71%)		2.04 (1.65, 2.54)
Arthritis	145 (38%)		951 (31%)		1.40 (1.12, 1.74)
Back Pain	132 (35%)		817 (27%)		1.44 (1.15, 1.81)
Heart disease	118 (31%)		916 (29%)		1.04 (0.83, 1.31)
Lung disease	79 (21%)		462 (15%)		1.46 (1.12, 1.91)
Cancer	39 (10%)		520 (17%)		0.57 (0.41, 0.80)
Depression/Anxiety	84 (22%)		396 (13%)		1.93 (1.48, 2.51)
Diabetes Mellitus	90 (24%)		353 (11%)		2.35 (1.81, 3.05)
Stroke	31 (8%)		122 (4%)		2.12 (1.41, 3.19)
≥ three chronic conditions	71 (19%)		242 (8%)		1.82 (0.46, 2.26)

^a^Measures the odds of each variable if the participant attends the public hospital versus the private hospital

^b^Not comparable between hospitals due to differences in coding methods

For nearly all other variables there were significant differences between respondents from the two hospitals. Participants attending the public hospital were much less likely to speak English at home (OR 0.11, 95% Confidence Interval (CI) 0.08–0.14), to have private health insurance (39.12, 29.95–51.09), to have left school before completion (3.90, 3.10–4.90), smoke (2.75, 1.81–4.18), be overweight or obese (2.59, 1.97–3.40), not participate in at least 2.5 h of physical activity per week (2.04, 1.65–2.54) and to be on a very low income (4.15, 3.30–5.23). Participants from the public hospital were almost 9 times more likely (8.98, 6.24–12.93) to have reported attending an emergency department on a previous occasion in the last 12 months. Compared with the private hospital sample, participants from the public hospital were more likely to have diabetes (2.35, 1.81–3.05), have had a stroke (2.12, 1.41–3.19), or experience anxiety or depression (1.93, 1.48–2.51). Prevalence of back pain, arthritis and heart disease were also significantly higher in the public hospital sample.

Table 2 compares health literacy scores by public versus private hospital, and the odds that attendance at the private hospital (reference variable) is likely to result in higher HLQ domain scores even when adjusted for variables known to be related to health literacy and use of hospital services. Unadjusted and adjusted OR are presented, adjusted for age, language, education and income. Participants attending the private hospital had higher health literacy. The exception was *Appraisal of health information*, where the scores for all participants was low independent of hospital. When adjusted for age, language, education, and income, patients attending the public hospital were still more likely to report lower scores (where the confidence interval did not include 1) for *Healthcare provider support* (adjusted OR 0.57, CI 0.44–0.72), *Actively managing my health* (0.67, 0.52–0.86), *Social support for Health* (0.66, 0.52–0.84), *Actively engaging with healthcare* 0.77, 0.64–0.93), *Navigating the healthcare system* (0.65, 0.53–0.79), and *Ability to find good health information* (0.77, 0.64–0.93), than those attending the private hospital.

**Table 2:** Relationship between Health Literacy Questionnaire mean domain scores by hospital of attendance, with unadjusted and adjusted odds ratios showing the difference in scale scores adjusted for variables known to be related to health literacy and use of hospital services

	Public Hospital (*N* = 384) Mean (SD)	Private (*N* = 3121) Mean (SD)	Unadjusted^c^	Adjusted Odds Ratio^c, d^
Healthcare provider support^a^	**3.13 (0.57)**	**3.35 (0.48)**	0.40 (0.32, 0.49)	0.57 (0.44, 0.72)
Having sufficient information^a^	**2.97 (0.52)**	**3.07 (0.48)**	0.59 (0.47, 0.74)	0.78 (0.61, 1.01)
Actively managing health^a^	**2.93 (0.52)**	**3.07 (0.49)**	0.54 (0.43, 0.67)	0.67 (0.52, 0.86)
Social support for health^a^	**3.11 (0.55)**	**3.26 (0.49)**	0.53 (0.43, 0.66)	0.66 (0.52, 0.84)
Active appraisal of health information^a^	2.82 (0.52)	2.85 (0.53)	0.87 (0.71, 1.07)	1.04 (0.82, 1.33)
Active engagement with healthcare^b^	**3.82 (0.78)**	**4.07 (0.58)**	0.52 (0.44, 0.61)	0.77 (0.64, 0.93)
Navigating the healthcare system^b^	**3.63 (0.75)**	**3.91 (0.57)**	0.45 (0.38, 0.54)	0.65 (0.53, 0.79)
Ability to find good health information^b^	**3.56 (0.80)**	**3.85 (0.62)**	0.51 (0.43, 0.59)	0.77 (0.64, 0.93)
Understanding health information well enough^b^	**3.85 (0.78)**	**4.11 (0.58)**	0.51 (0.43, 0.60)	0.87 (0.71, 1.05)

^a^Scale range 0–4, higher score indicates greater ability or more support

^b^Scale range 0–5, higher score indicates greater ability or more support

^c^Reference category = private hospital

^d^Adjusted Odds Ratio. Adjusted for age (< 65, ≥65), education (did not complete secondary school, completed secondary school or above), language (English, non-English), income (<$30,000, ≥$30,000)

Items in bold indicate significance at the *p* = 0.05 level

Table 3 provides a summary of the significant associations across both hospital populations between having lower scores on the HLQ and poorer health and/or health related behaviours. Both public and private hospital participants who were smokers, and/or those who participated in less than 2.5 h of physical activity per week reported lower scores for *Actively managing my health*, compared to those who did not smoke and/or participated in at least 2.5 h of physical activity per week. Compared to participants reporting less than three chronic conditions, those with three or more conditions reported lower health literacy scores for *Ability to find good health information*, and *Understanding information well enough to know what to do*. Having depression and/or anxiety was associated with lower scores for *Social support for health* and *Ability to actively engage with healthcare providers.* The full details of the associations between health literacy and behavioural and health factors are presented as Additional files 1 and 2.

**Table 3:** Summary of significant associations using effect sizes (ES) across both hospitals between lower Health Literacy Questionnaire scores and poorer health and/or health related behaviours

	Healthcare provider support	Having sufficient information	Actively managing health	Social support for health	Active appraisal	Active engagement with healthcare	Navigating the healthcare system	Ability to find good health information	Understanding health information
Current smoker			Private ES 0.28 Public ES 0.27						
Physical Activity < 2.5 h/week			Private ES 0.50 Public ES 0.27						
Depression/ Anxiety				Private ES 0.27 Public ES 0.39		Private ES 0.15 Public ES 0.30			
≥3 chronic conditions								Private ES 0.26 Public ES 0.25	Private ES 0.18 Public ES 0.20

*Effect size (ES) calculated using Cohen’s d for standardised difference in means. Interpretation of ES: *“small” ES > 0.20–0.50, **“medium” ES 0.50–0.80

## Discussion

We found that compared with participants who had received care at the private hospital, those who had received care at the public hospital had significantly lower health literacy across eight of the nine domains of the HLQ. The only domain that did not differ between hospital samples was *Active appraisal of health information.* After adjustment for differences in age, education, language and income, participants who attended the public hospital were still more likely to report lower scores than private hospital patients for five domains that measure an individual’s lived experience of interacting with the health system: *Feeling supported by healthcare providers*, *Actively managing health*, *Active engagement with healthcare*, *Navigating the healthcare system* and *Ability to find good health information*. This suggests that the private hospital may possess organisational characteristics (environment, structure, values, practices and/or workforce competencies) that may result in a greater level of health literacy responsive service delivery than the public hospital. Despite these differences in health literacy responsiveness, we found shared patterns of lower health literacy associated with individuals presenting with three or more chronic health conditions, those who currently smoke, those who do not participate in regular physical activity, and those with anxiety and/or depression exhibited across both hospitals.

As the HLQ in part aims to measure an individual’s experience of receiving care, the differences in the health literacy of these two hospital populations, independent of differences in socioeconomic position and first language, suggest that the impact that these two hospitals have on their population’s health literacy is different. The reasons for this are likely to be complex and multifactorial. In part, it may be explained by the more demanding communication effort required of the public hospital and its health professionals. While our analysis controlled for differences in language, income and education, we did not control for the much greater disease burden or cultural diversity (independent of language spoken) in the public hospital setting (as demonstrated by the odds ratios in Table 1). These added complexities, coupled with lower education and income levels, present a challenging combination of factors known to impact on an individual’s health literacy [32].

In addition, differences in health literacy between the private and public hospital patients may be a reflection of how differences in funding arrangements drive care. Private and public hospitals do not have the same operational environments or demand pressures. Public hospitals provide care according to need, and while demand for hospital services are influenced by a range of factors, an ageing population [33] and growth in chronic and complex diseases [34] has led to public hospital overcrowding, bed blockages, lengthening surgical waiting lists and problems with timely access to emergency treatment and unplanned admissions both in Australia and internationally [35, 36]. Private hospitals are not subject to these same demand pressures. In addition, as private enterprises, private hospitals are reliant on establishing and maintaining market reputation. They need to provide high quality customer service in order to maintain loyalty, retention and demand from their paying consumer and private insurers [37]. The private health model provides the consumer with control over almost all aspects of their health care decision making. Retention has been shown to be more closely related to the functional aspects of care-giving (how it is done) rather than the technical aspects (what is done) [38]. Building patient rapport, taking time and care to ensure patients understand their health condition and know where to go for more information, and creating an easily navigable hospital with good signage, all fall under functional aspects of health care delivery. Therefore, while the technical aspects of clinical care may not differ between public and private hospitals, private hospitals may be better placed to put greater emphasis on the functional aspects of care delivery which will in turn impact on an individual’s health literacy.

We found that individuals with multiple health conditions reported similarly low scores at both hospitals across domains that measure how confident an individual is in finding and understanding health information. Patients at both hospitals who had three or more chronic conditions were more likely to report challenges around finding, understanding and managing information in relation to their multiple conditions. Given that most information available to patients is based on single disease guidelines, this finding is not surprising. People with multiple conditions may find that two or more of their conditions have discordant management guidelines and treatment recommendations. For example, an individual with diabetes, renal failure and osteoarthritis may read that non-steroidal anti-inflammatory drugs (NSAIDs) will help reduce joint pain and inflammation. However, NSAIDs should not be used in patients with kidney failure, and may lead to further diabetes-related complications and even death [39]. With up to 65% of individuals with chronic conditions having multiple comorbid diseases, the problem of single disease guidelines presents a significant challenge to an individual’s cognitive load, and their capacity to be sufficiently health literate about their conditions [40]. In this way, individuals with the greatest health care needs are most likely to have the lowest ability to comprehend the information required to manage their conditions, and to navigate and function effectively within the healthcare system [40, 41].

In 2005, Bernhardt and colleagues identified that ‘in order for individuals to effectively access, understand and apply received health messages … they must be motivated to receive and process the information’ [42]. Individuals who are able to independently take actions to manage their health and healthcare have better health outcomes [43]. We found that current smokers and study participants with low levels of weekly physical activity at both hospitals had lower health literacy for the ‘*Actively managing health’* domain. It is possible that Individuals who scored low on this domain may not see health as their responsibility and have lower levels of motivation to understand their condition in order to self-manage.

Consistent with previous research, participants experiencing depression and/or anxiety reported additional health literacy challenges when compared to participants with other chronic conditions [28]. Across both public and private hospitals, participants with depression and/or anxiety reported lower scores for *Social support for health* and *Active engagement with healthcare providers*. A recent diagnosis of some chronic diseases is associated with an increased risk for the development of anxiety and depression [44–46], and it is shown that individuals with a chronic disease and low functional health literacy have higher levels of anxiety than individuals with adequate health literacy [47]. In some cases, having a chronic disease and depression/anxiety, and feeling socially isolated, has been shown to significantly increase mortality risk [48, 49]. As having depression and/or anxiety in this study was related to *Social support for health* and *Active engagement with health providers,* greater efforts to engage and link patients with appropriate support may lead to improved health outcomes for these patients.

This study has a number of limitations. The samples from the two hospitals differed in size which may increase the potential for confounding. However, we had sufficient power to address the research questions, and the distribution of data was similar across the two datasets. There is also the possibility that differences in the health literacy of patients attending the two hospitals relate to the types of patients attracted to each hospital. Although we did include potential confounders in the statistical model, it is possible that the observed differences in health literacy found between the two public and the private hospital relate to other unmeasured characteristics linked to the type of people attracted to each hospital. Our findings are also specific to the two hospitals studied, each of which should not be regarded as representative of all public or private hospitals in Australia. Variation in the sociodemographic characteristics of the people residing in the geographical location of the hospitals may generate different results given that health literacy is strongly associated with socioeconomic status. That said, earlier work in Victoria across eight disparate organisations found that the HLQ provides unbiased mean estimates of group differences across key demographic indicators, suggesting that the findings are not likely to be the result of measurement bias [27].

Despite similar study protocols, the response rate for the survey was substantially higher from participants who were hospitalised in the private hospital (33% vs. 13%). The lower response rate in the public hospital sample is likely to be related to their overall lower health literacy, as those who return surveys are more likely to have higher socioeconomic indicators and higher health literacy [50–53]. An underrepresentation of individuals with low and very low health literacy may also have led to an underestimate of the differences between hospital samples and the relationship between socioeconomic factors, chronic disease and/or health behaviours and health literacy. Future research may be strengthened through application of sensitivity analyses exploring the impact of the non-English speaking respondents in the samples.

This study has several implications for future research. To date, most interventions designed to improve health literacy have focused on health literacy as an individual attribute. They have therefore tried to address deficits by simplifying written health information or improving the composition or method of delivery of messages (including signage, brochures and instructional material) so that it is more user-friendly [32, 54]. Our findings further support the conceptualisation of health literacy not as an individual attribute, but as an interactive process, influenced and mediated by supportive relationships with health providers and the health system at large. Recent research has identified attributes that make for a health literacy responsive organisation [13, 15]. There is now a need for research that determines whether development of these attributes within an organisation leads to improvements in the health literacy of service users.

Additionally, we found that irrespective of the service delivery model (public or private), the presence of three or more chronic diseases, anxiety and/or depression, smoking, and/or low levels of physical activity appear to be associated with lower levels of some elements of health literacy. If this finding is substantiated in future studies, research that evaluates interventions to improve health literacy for these individuals may result in improvements in access, understanding and use of health information and services (i.e., health literacy).

## Conclusion

The observation of lower health literacy of patients who receive their care at a public hospital compared to a private hospital, even after adjusting for sociodemographic differences suggests that private hospitals may possess organisational attributes or characteristics (environment, structure, values, practices and/or workforce competencies) that result in improved health literacy responsiveness.

## Additional files

Additional file 1: **Table S1.** Association between mean (SD) Health Literacy Questionnaire domain scores and chronic conditions using effect sizes, by hospital of attendance. (DOCX 52 kb)

Additional file 2: **Table S2.** Association between mean (SD) Health Literacy Questionnaire domain scores and health related behaviour using effect sizes, by hospital of attendance. (DOCX 49 kb)

## Abbreviations

CI: Confidence Interval
ED: Emergency Department
ES: Effect Sizes
HLQ: Health Literacy Questionnaire
OR: Odds Ratios

## References

[1] World Health Organization (1998). Health promotion glossary. Health Promot Int.

[2] Williams MV (1998). Relationship of functional health literacy to patients' knowledge of their chronic disease: a study of patients with hypertension and diabetes. Arch Intern Med.

[3] Schillinger D (2002). Association of health literacy with diabetes outcomes. JAMA.

[4] Gazmararian JA (2003). Health literacy and knowledge of chronic disease. Patient Educ Couns.

[5] Williams MV (1998). Inadequate literacy is a barrier to asthma knowledge and self-care. Chest J.

[6] Souza JG (2014). Functional health literacy and glycaemic control in older adults with type 2 diabetes: a cross-sectional study. BMJ Open.

[7] Gazmararian JA (2006). Factors associated with medication refill adherence in cardiovascular-related diseases: a focus on health literacy. J Gen Intern Med.

[8] Omachi TA (2013). Lower health literacy is associated with poorer health status and outcomes in chronic obstructive pulmonary disease. J Gen Intern Med.

[9] Baker DW (1998). Health literacy and the risk of hospital admission. J Gen Intern Med.

[10] Baker DW (2002). Functional health literacy and the risk of hospital admission among Medicare managed care enrollees. Am J Public Health.

[11] Peterson PN (2011). Health literacy and outcomes among patients with heart failure. JAMA.

[12] Pleasant A, Kuruvilla S (2008). A tale of two health literacies: public health and clinical approaches to health literacy. Health Promot Int.

[13] Trezona A, Dodson S, Osborne RH (2017). Development of the organisational health literacy responsiveness (Org-HLR) framework in collaboration with health and social services professionals. BMC Health Serv Res.

[14] Wynia MK, Osborn CY (2010). Health literacy and communication quality in health care organizations. J Health Commun.

[15] Brach C (2012). Attributes of a health literate organization.

[16] Brega AG (2015). *Health Literacy Universal Precautions Toolkit*.

[17] Australian Commission on Quality and Safety in Health Care (2014). *Health Literacy: Taking action to improve safety and quality*.

[18] Hurley J (2002). Parallel private health insurance in Australia: a cautionary tale and lessons for Canada.

[19] Bourne A., P.S., Jessup R., Staples M., Beauchamp A., Buchbinder R.,, *Health literacy profile of recently hospitalised patient in the private hospital setting: A cross sectional study.* BMC health services research (under review), 2017. 10.1186/s12913-018-3697-2 PMC6247774 30458773

[20] Jessup RL (2017). Health literacy of recently hospitalised patients: a cross-sectional survey using the health literacy questionnaire (HLQ). BMC Health Serv Res.

[21] State Government of Victoria. *Index of Relative Socioeconomic Disadvantage*. 2015 [cited 2015 February 23].

[22] Beauchamp A, Batterham RW, Dodson S, Astbury B, Elsworth GR, McPhee C, Osborne RH. Systematic development and implementation of interventions to OPtimise Health Literacy and Access (Ophelia). BMC public health. 2017;17(1):230. 10.1186/s12889-017-4147-5 PMC5335493 28253883

[23] Hawkins M, et al. The health literacy questionnaire (HLQ) at the patient-clinician interface: what do patients and clinicians really mean by their HLQ scores? BMC Health Serv Res. 2017; 10.1186/s12913-017-2254-8 PMC5408483 28449680

[24] Kolarcik P, et al. Structural properties and psychometric improvements of the health literacy questionnaire in a Slovak population. International Journal of Public Health. 2017:1–14. 10.1007/s00038-017-0945-x 28258403

[25] Nolte S, Osborne RH, Dwinger S, Elsworth GR, Conrad ML, Rose M, Zill JM. German translation, cultural adaptation, and validation of the Health Literacy Questionnaire (HLQ). PloS one. 2017;12(2):e0172340. 10.1371/journal.pone.0172340 PMC5325258 28234987

[26] Osborne RH (2013). The grounded psychometric development and initial validation of the health literacy questionnaire (HLQ). BMC Public Health.

[27] Elsworth GR, Beauchamp A, Osborne RH (2016). Measuring health literacy in community agencies: a Bayesian study of the factor structure and measurement invariance of the health literacy questionnaire (HLQ). BMC Health Serv Res.

[28] Maindal HT (2016). Cultural adaptation and validation of the health literacy questionnaire (HLQ): robust nine-dimension Danish language confirmatory factor model. SpringerPlus.

[29] IBM Corp, IBM SPSS Statistics for Windows, Version 22.0*.* Armonk, NY, released 2013. IBM Corp**.**

[30] StataCorp (2015). *Stata Statistical Software: Release 14*.

[31] Cohen J. Statistical power analysis for the behavioral sciences. Hilsdale. NJ: Lawrence Earlbaum Associates 2. 1988.

[32] Berkman ND (2011). Low health literacy and health outcomes: an updated systematic review. Ann Intern Med.

[33] Walker A. *Australia's ageing population*. Vol. no. 27. Canberra, A.C.T: National Centre for social and economic modelling, University of Canberra; 1998.

[34] Australian Institute of Health and Welfare (2012). *Risk factors contributing to chronic disease*.

[35] Sammut J (2009). Why public hospitals are overcrowded: ten points for policymakers.

[36] Lowthian JA (2012). Demand at the emergency department front door: 10-year trends in presentations. Med J Aust.

[37] Boshoff C, Gray B (2004). The relationships between service quality, customer satisfaction and buying intentions in the private hospital industry. South African Journal of Business Management.

[38] Shemwell DJ, Yavas U, Bilgin Z (1998). Customer-service provider relationships: an empirical test of a model of service quality, satisfaction and relationship-oriented outcomes. Int J Serv Ind Manag.

[39] Boyd CM, Fortin M (2010). Future of multimorbidity research: how should understanding of multimorbidity inform health system design?. Public Health Rev.

[40] Wolff JL, Starfield B, Anderson G (2002). Prevalence, expenditures, and complications of multiple chronic conditions in the elderly. Arch Intern Med.

[41] Parker RM, Ratzan SC, Lurie N (2003). Health literacy: a policy challenge for advancing high-quality health care. Health Aff.

[42] Schwartzberg JG, VanGeest JB, Wang CC (2005). Understanding health literacy.

[43] Hibbard JH, Greene J (2013). What the evidence shows about patient activation: better health outcomes and care experiences; fewer data on costs. Health Aff.

[44] Hackett ML (2005). Frequency of depression after stroke a systematic review of observational studies. Stroke.

[45] Kotila M (1998). Depression after stroke results of the finnstroke study. Stroke.

[46] Glassman AH (2002). Sertraline treatment of major depression in patients with acute MI or unstable angina. JAMA.

[47] Rowlands GP (2013). Characteristics of people with low health literacy on coronary heart disease GP registers in South London: a cross-sectional study. BMJ Open.

[48] Bush DE (2001). Even minimal symptoms of depression increase mortality risk after acute myocardial infarction. Am J Cardiol.

[49] Friedmann E (2006). Relationship of depression, anxiety, and social isolation to chronic heart failure outpatient mortality. American heart journal.

[50] Fredrickson DD (2005). Optimal design features for surveying low-income populations. J Health Care Poor Underserved.

[51] Gibson PJ (1999). Increasing response rates for mailed surveys of Medicaid clients and other low-income populations. Am J Epidemiol.

[52] Beebe TJ (2005). Increasing response rates in a survey of Medicaid enrollees: the effect of a prepaid monetary incentive and mixed modes (mail and telephone). Med Care.

[53] Barber MN (2009). Up to a quarter of the Australian population may have suboptimal health literacy depending upon the measurement tool: results from a population-based survey. Health Promot Int.

[54] Pignone M (2005). Interventions to improve health outcomes for patients with low literacy. J Gen Intern Med.

