# Supplementary material for: Differences in health literacy profiles of patients admitted to a public and a private hospital in Melbourne, Australia
Source: BMC Health Serv Res. 2018 Feb 22;18:134. doi: 10.1186/s12913-018-2921-4 (PMC5824469; doi:10.1186/s12913-018-2921-4)
Supplement: Supplementary file 1 — Table S1. Association between mean (SD) Health Literacy Questionnaire domain scores and chronic conditions using effect sizes, by hospital of attendance. (DOCX 52 kb) [file 12913_2018_2921_MOESM1_ESM.docx]

Additional file 1: Table S1 Association between Health Literacy Questionnaire domain scores and chronic conditions, by hospital of attendance

|  |  | **Healthcare provider support**^#^ | | **Having sufficient information**^#^ | | **Actively managing health**^#^ | | **Social support for health**^#^ | | **Active appraisal of health information**^#^ | | **Active engagement with healthcare**^##^ | | **Navigating the healthcare system**^##^ | | **Ability to find good health information**^##^ | | **Understanding health information**^##^ | |
| --- | --- | --- | --- | --- | --- | --- | --- | --- | --- | --- | --- | --- | --- | --- | --- | --- | --- | --- | --- |
|  |  | Public | Private | Public | Private | Public | Private | Public | Private | Public | Private | Public | Private | Public | Private | Public | Private | Public | Private |
| Depress./ anxiety | Yes (Public n=84 Private n= 393) | 3.09  *(0.63)* | 3.36  *(0.47)* | 2.92  *(0.59)* | 3.02  *(0.48)* | 2.89  *(0.58)* | **2.99**  ***(0.46)*** | **2.95**  ***(0.68)*** | **3.14**  ***(0.53)*** | 2.81  *(0.53)* | 2.82  *(0.50)* | **3.64**  ***(0.88)*** | **4.00**  ***(0.62)*** | 3.56  *(0.83)* | **3.83**  ***(0.59)*** | 3.45  *(0.85)* | **3.76**  ***(0.66)*** | 3.72  *(0.85)* | **4.02**  ***(0.62)*** |
|  | No (Public n= 296, Private n= 2644) | 3.14  *(0.54)* | 3.34  *(0.48)* | 2.98  *(0.49)* | 3.08  *(0.47)* | 2.94  *(0.50)* | **3.09**  ***(0.49)*** | **3.15**  ***(0.49)*** | **3.27**  ***(0.48)*** | 2.82  *(0.52)* | 2.85  *(0.54)* | **3.87**  ***(0.74)*** | **4.08**  ***(0.57)*** | 3.65  *(0.73)* | **3.92**  ***(0.56)*** | 3.59  *(0.78)* | **3.86**  ***(0.61)*** | 3.89  *(0.76)* | **4.13**  ***(0.57)*** |
|  | Effect size (95% CI) | -0.09  (-0.33,  0.15 | 0.04  (-0.07,  0.14) | -0.12  (-0.36,  0.12) | -0.13  (-0.24,  -0.02 | -0.10  (-0.34,  0.14 | **-0.20***  **(-0.31,**  **-0.09)** | **-0.39***  **(-0.63,**  **-0.14)** | **-0.27***  **(-0.37,**  **-0.16)** | -0.03  (-0.27,  0.21) | -0.05  (-0.15,  0.06) | **-0.30***  **(-0.54,**  **-0.06)** | **-0.15**  **(-0.26,**  **-0.04)** | -0.12  (-0.37,  0.12) | **-0.16**  **(-0.27,**  **-0.05)** | -0.17  (-0.41,  0.07) | **-0.16**  **(-0.27,**  **-0.06)** | -0.22  (-0.46,  0.03) | **-0.18**  **(-0.29,**  **-0.07)** |
| Lung Condition | Yes (Public n=79 Private n= 458) | 3.11  *(0.52)* | **3.39**  ***(0.48)*** | 2.90  *(0.57)* | 3.06  *(0.49)* | 2.93  *(0.53)* | 3.05  *(0.52)* | 3.05  *(0.55)* | 3.23  *(0.53)* | 2.81  *(0.53)* | 2.84  *(0.56)* | 3.75  *(0.77)* | 4.07  *(0.59)* | 3.59  *(0.84)* | 3.89  *(0.58)* | 3.47  *(0.86)* | 3.82  *(0.66)* | 3.82  *(0.86)* | 4.09  *(0.59)* |
|  | No  (Public n=304,  Private n= 2581) | 3.13  *(0.58)* | **3.34**  ***(0.48)*** | 2.98  *(0.50)* | 3.08  *(0.47)* | 2.93  *(0.52)* | 3.08  *(0.48)* | 3.12  *(0.54)* | 3.26  *0.48)* | 2.82  *0.52)* | 2.84  *0.53)* | 3.84  *0.78)* | 4.07  *0.57)* | 3.65  *0.73)* | 3.91  *0.56)* | 3.58  *0.78)* | 3.85  *0.61)* | 3.86  *0.76)* | 4.12  *0.57)* |
|  | Effect size (95% CI) | -0.02  (-0.27,  0.22) | **0.11**  **(0.01,**  **0.20)** | -0.15  (-0.40,  0.10) | -0.04  (-0.14,  0.06) | 0.00  (-0.25,  0.24) | -0.05  (-0.15,  0.05) | -0.13  (-0.38,  0.12) | -0.06  (-0.16,  0.04) | -0.02  (-0.26,  0.23) | 0.00  (-0.10,  0.10) | -0.11  (-0.36,  0.14) | -0.01  (-0.11,  0.09) | -0.08  (-0.33,  0.17) | -0.04  (-0.14,  0.06) | -0.13  (-0.38,  0.12) | -0.06  (-0.16,  0.04) | -0.05  (-0.30,  0.20) | -0.04  (-0.14,  0.06) |
| Cancer | Yes (Public n=39,  Private n=513) | 3.05  *(0.57)* | 3.41  *(0.48)* | 3.01  *(0.59)* | 3.08  *(0.50)* | 2.84  *(0.51)* | 3.09  *(0.49)* | 3.10  *(0.55)* | **3.34**  ***(0.49)*** | 2.77  *(0.54)* | 2.86  *(0.54)* | 3.79  *(0.76)* | 4.08  *(0.60)* | 3.55  *(0.79)* | 3.93  *(0.57)* | 3.51  *(0.79)* | 3.80  *(0.63)* | 3.74  *(0.85)* | 4.09  *(0.59)* |
|  | No (Public n= 342,  Private n= 2526) | 3.13  *(0.56)* | 3.33  *(0.48)* | 2.96  *(0.51)* | 3.07  *(0.47)* | 2.94  *(0.52)* | 3.07  *(0.49)* | 3.11  *(0.55)* | **3.24**  ***(0.48)*** | 2.83  *(0.52)* | 2.84  *(0.53)* | 3.83  *(0.78)* | 4.07  *(0.57)* | 3.64  *(0.75)* | 3.91  *(0.56)* | 3.57  *(0.80)* | 3.86  *(0.62)* | 3.86  *(0.77)* | 4.12  *(0.57)* |
|  | Effect size (95% CI) | -0.16  (-0.49,  0.18) | 0.16  (0.06,  0.25) | 0.09  (-0.24,  0.42) | 0.03  (-0.07,  0.12) | -0.19  (-0.53  0.15) | 0.03  (-0.07  0.12) | -0.02  (-0.35  0.31) | **0.20***  **(0.11**  **0.30)** | -0.10  (-0.44  0.24) | 0.03  (-0.07  0.12) | -0.04  (-0.38  0.29) | 0.02  (-0.07  0.12) | -0.12  (-0.45  0.22) | 0.03  (-0.06  0.13) | -0.08  (-0.41  0.26) | -0.08  (-0.18  0.01) | -0.15  (-0.48  0.18) | -0.05  (-0.15  0.04) |
|  |  |  |  |  |  |  |  |  |  |  |  |  |  |  |  |  |  |  |  |
| Diabetes Mellitus | Yes (Public n=90,  Private n=350) | 3.22  *(0.50)* | 3.39  *(0.46)* | 3.05  *(0.48)* | 3.06  *(0.49)* | 2.97  *(0.42)* | 3.03  *(0.49)* | 3.13  *(0.46)* | 3.26  *(0.51)* | 2.80  *(0.51)* | 2.87  *(0.52)* | 3.85  *(0.78)* | 4.07  *(0.61)* | 3.61  *(0.78)* | 3.87  *(0.61)* | 3.49  *(0.88)* | **3.73**  ***(0.69)*** | 3.82  *(0.83)* | **3.99**  ***(0.63)*** |
|  | No (Public n=292, Private n=2688) | 3.10  *(0.58* | 3.34  *(0.48* | 2.94  *(0.52* | 3.08  *(0.47* | 2.92  *(0.55* | 3.08  *(0.49* | 3.10  *(0.57* | 3.26  *(0.48* | 2.83  *(0.52* | 2.84  *(0.53* | 3.81  *(0.78* | 4.07  *(0.57)* | 3.64  *(0.74)* | 3.92  *(0.56)* | 3.58  *(0.77)* | **3.86**  ***(0.61)*** | 3.86  *(0.76)* | **4.13**  ***(0.57)*** |
|  | Effect size (95% CI) | 0.21*  (-0.03,  0.45) | 0.11  (0.00,  0.22) | 0.22*  (-0.01,  0.46) | -0.04  (-0.15,  0.07) | 0.09  (-0.15,  0.33) | -0.09  (-0.21,  0.02) | 0.06  (-0.17,  0.30) | 0.01  (-0.10,  0.12) | -0.04  (-0.28,  0.19) | 0.06  (-0.05,  0.18) | 0.05  (-0.19,  0.29) | -0.01  (-0.12,  0.10) | -0.04  (-0.28,  0.20) | -0.08  (-0.19,  0.03) | -0.11  (-0.35,  0.13) | **-0.21***  **(-0.32,**  **-0.10)** | -0.04  (-0.28,  0.20) | **-0.25***  **(-0.36,**  **-0.13)** |
| Stroke | Yes (Public n= 31,  Private n= 119) | 3.23  *(0.47)* | 3.31  *(0.46)* | 3.03  *(0.45)* | 2.99  *(0.46)* | 2.90  *(0.60)* | 2.98  *(0.47)* | 3.23  *(0.45)* | 3.17  *(0.50)* | 2.77  *(0.54)* | **2.70**  ***(0.52)*** | 3.96  *(0.71)* | 4.04  *(0.54)* | 3.75  *(0.66)* | 3.90  *(0.55)* | 3.60  *(0.73)* | **3.72**  ***(0.63*** | 3.94  *(0.74)* | **3.96**  ***(0.56)*** |
|  | No  (Public n=351,  Private n= 2919) | 3.12  *(0.57)* | 3.35  *(0.48)* | 2.96  *(0.52)* | 3.08  *(0.47)* | 2.93  *(0.51)* | 3.08  *(0.49)* | 3.10  *(0.55)* | 3.26  *(0.49)* | 2.82  *(0.52)* | **2.85**  ***(0.53)*** | 3.81  *(0.78)* | 4.07  *(0.58)* | 3.62  *(0.76)* | 3.91  *(0.57)* | 3.56  *(0.80)* | **3.85**  ***(0.62)*** | 3.84  *(0.78)* | **4.12**  ***(0.58)*** |
|  | Effect size (95% CI) | 0.21*  (-0.16,  0.58) | -0.09  (-0.27,  0.10) | 0.14  (-0.23,  0.51) | -0.18  (-0.37,  0.00) | -0.07  (-0.44  0.30) | -0.19  (-0.37,  -0.01) | 0.24  (-0.13,  0.60) | -0.19  (-0.37,  -0.01) | -0.11  (-0.47,  0.26) | **-0.29***  **(-0.47,**  **-0.10)** | 0.20  (-0.17,  0.56) | -0.05  (-0.24,  0.13) | 0.17  (-0.20,  0.53) | -0.03  (-0.21,  0.16) | 0.06  (-0.31,  0.42) | **-0.21***  **(-0.40,**  **-0.03)** | 0.13  (-0.24,  0.50) | **-0.27***  **(-0.45,**  **-0.08)** |
| Heart Condition | Yes (Public n= 117  Private n= 906) | **3.19**  ***(0.57)*** | **3.39**  ***(0.47)*** | 2.96  *(0.55)* | 3.06  *(0.49)* | **3.02**  ***(0.54)*** | 3.07  *(0.49)* | 3.15  *(0.53)* | 3.28  *(0.50)* | 2.83  *(0.55)* | 2.82  *(0.54)* | 3.71  *(0.88)* | 4.07  *(0.57)* | 3.52  *(0.82)* | 3.91  *(0.58)* | **3.41**  ***(0.92)*** | **3.76**  ***(0.65)*** | **3.71**  ***(0.89)*** | **4.07**  ***(0.60)*** |
|  | No (Public n=266,  Private n=2111) | **3.10**  ***(0.56)*** | **3.33**  ***(0.48)*** | 2.97  *(0.50)* | 3.08  *(0.47)* | **2.89**  ***(0.51)*** | 3.08  *(0.49)* | 3.09  *(0.55)* | 3.25  *(0.48)* | 2.82  *(0.50)* | 2.86  *(0.53)* | 3.87  *(0.73)* | 4.07  *(0.58)* | 3.68  *(0.71)* | 3.91  *(0.56)* | **3.63**  ***(0.73)*** | **3.88**  ***(0.60)*** | **3.91**  ***(0.72)*** | **4.13**  ***(0.56)*** |
|  | Effect size (95% CI) | **0.16**  **(0.16,**  **0.11)** | **0.12**  **(0.12,**  **0.04)** | -0.02  (-0.24,  0.20) | -0.04  (-0.12,  0.04) | **0.26***  **(0.04,**  **0.47)** | -0.02  (-0.10,  0.06) | 0.10  (-0.12,  0.32) | 0.06  (-0.02,  0.14) | 0.03  (-0.19,  0.25) | -0.07  (-0.15,  0.01) | -0.21*  (-0.42,  0.01) | 0.01  (-0.07,  0.08) | -0.21*  (-0.43,  0.01) | 0.00  (-0.08,  0.08) | **-0.28***  **(-0.50,**  **-0.06)** | **-0.20***  **(-0.28,**  **-0.12)** | **-0.26***  **(-0.48,**  **-0.04)** | **-0.10**  **(-0.18,**  **-0.03)** |
| Back Pain | Yes (Public n=131,  Private n=808) | 3.11  *(0.55)* | 3.37  *(0.47)* | 2.94  *(0.52)* | 3.04  *(0.47)* | 2.89  *(0.51)* | 3.03  *(0.47)* | 3.05  *(0.58)* | 3.22  *(0.53)* | 2.79  *(0.54)* | 2.83  *(0.52)* | **3.77**  ***(0.80)*** | 4.07  *(0.58)* | 3.57  *(0.81)* | 3.89  *(0.58)* | **3.42**  ***(0.86)*** | **3.80**  ***(0.63)*** | 3.75  *(0.82)* | 4.10  *(0.59)* |
|  | No (Public n= 251,  Private = 2231) | 3.13  *(0.57)* | 3.34  *(0.48)* | 2.98  *(0.51)* | 3.09  *(0.47)* | 2.95  *(0.52)* | 3.09  *(0.49)* | 3.14  *(0.52)* | 3.27  *(0.47)* | 2.84  *(0.51)* | 2.85  *(0.53)* | **3.85**  ***(0.77)*** | 4.07  *(0.57)* | 3.67  *(0.72)* | 3.92  *(0.56)* | **3.63***  ***(0.76)*** | **3.86**  ***(0.62)*** | 3.90  *(0.76)* | 4.12  *(0.57)* |
|  | Effect size (95% CI) | -0.05  (-0.26  0.16) | 0.07  (-0.01,  0.15) | -0.07  (-0.28,  0.15) | -0.11  (-0.19,  -0.03) | -0.11  (-0.32,  0.10) | -0.11  (-0.19,  -0.03) | -0.17  (-0.38,  0.04) | -0.11  (-0.19,  -0.02) | -0.10  (-0.31,  0.11) | -0.04  (-0.11,  -0.01) | **-0.12**  **(-0.32,**  **-0.09)** | 0.04  (0.10,  0.07) | -0.13  (-0.34,  0.08) | -0.04  (-0.12,  0.04) | **-0.26***  **(-0.47,**  **-0.05**) | **-0.11**  **(-0.19,**  **-0.03)** | -0.20*  (-0.41,  0.02) | -0.03  (-0.11,  0.05) |
| Arthritis | Yes (Public n=144, Private n=943) | 3.19  *(0.53)* | 3.35  *(0.47)* | 3.02  *(0.49)* | 3.06  *(0.48)* | **3.00**  ***(0.50)*** | 3.07  *(0.47)* | 3.14  *(0.51)* | 3.25  *(0.50)* | 2.88  *(0.55)* | 2.85  *(0.52)* | 3.81  *(0.81)* | 4.06  *(0.59)* | 3.67  *(0.76)* | 3.90  *(0.57)* | 3.49  *(0.84)* | 3.78  *(0.66)* | 3.77  *(0.83)* | 4.07  *(0.61)* |
|  | No  (Public n=238, Private n=2095) | 3.09  *(0.58)* | 3.34  *(0.48)* | 2.93  *(0.53)* | 3.08  *(0.47)* | **2.89**  ***(0.53)*** | 3.08  *(0.50)* | 3.09  *(0.57)* | 3.26  *(0.48)* | 2.78  *(0.50)* | 2.84  *(0.54)* | 3.83  *(0.76)* | 4.08  *(0.57)* | 3.61  *(0.75)* | 3.91  *(0.56)* | 3.60  *(0.77)* | 3.87  *(0.60)* | 3.90  *(0.75)* | 4.13  *(0.56)* |
|  | Effect size (95% CI) | 0.19  (-0.02,  0.39 | 0.02  (-0.06,  0.10) | 0.16  (-0.05,  0.37) | -0.05  (-0.13,  0.02) | **0.21***  **(0.01,**  **0.42)** | -0.03  (-0.10,  0.05) | 0.10  (-0.11,  0.31) | -0.02  (-0.09,  0.06) | 0.19  (-0.02,  0.40) | 0.01  (-0.07,  0.09) | -0.03  (-0.23,  0.18) | -0.03  (-0.11,  0.05) | 0.08  (-0.13,  0.28) | -0.02  (-0.10,  0.05) | -0.14  (-0.35,  0.06) | -0.15  (-0.23,  -0.07) | -0.17  (-0.38,  0.04) | -0.10  (-0.18,  -0.02) |
| 3 or more chronic conditions | Yes (Public n=150,  Private n=814) | 3.17  *(0.55)* | **3.38**  ***(0.47)*** | 2.99  *(0.53)* | **3.03**  ***(0.49)*** | 2.97  *(0.49)* | 3.05  *(0.48)* | 3.07  *(0.55)* | 3.23  *(0.52)* | 2.85  *(0.04)* | 2.83  *(0.52)* | 3.75  *(0.83)* | **4.03**  ***(0.60*** | 3.57  *(0.80)* | **3.86**  ***(0.59)*** | **3.44**  ***(0.89)*** | **3.73**  ***(0.67)*** | **3.75**  ***(0.87)*** | **4.03**  ***(0.61)*** |
|  | No (Public n=232,  Private = 2277) | 3.10  *(0.57)* | **3.33**  ***(0.48)*** | 2.95  *(0.51)* | **3.09**  ***(0.47)*** | 2.90  *(0.54)* | 3.09  *(0.49)* | 3.13  *(0.54)* | 3.26  *(0.47)* | 2.80  *(0.50)* | 2.85  *(0.53)* | 3.87  *(0.74)* | **4.08**  ***(0.57)*** | 3.67  *(0.71)* | **3.92**  ***(0.55)*** | **3.64**  ***(0.73)*** | **3.89**  ***(0.60)*** | **3.91**  ***(0.71)*** | **4.14**  ***(0.56)*** |
|  | Effect size(95% CI) | 0.13  (-0.07,  0.34) | **0.10**  **(0.02,**  **0.18)** | 0.08  (-0.13,  0.29) | **-0.11**  **(-0.19,**  **-0.03)** | 0.14  (-0.07,  0.35) | -0.08  (-0.16,  0.00) | -0.10  (-0.31,  0.10) | -0.06  (-0.14,  0.02) | 0.11  (-0.10,  0.32) | -0.04  (-0.12,  0.04) | -0.15  (-0.36,  0.05) | **-0.09**  **(-0.17,**  **-0.01)** | -0.14  (-0.34,  0.07) | **-0.11**  **(-0.19,**  **-0.03)** | **-0.25***  **(-0.46,**  **-0.05)** | **-0.26***  **(-0.35,**  **-0.18)** | **-0.20***  **(-0.41,**  **0.01)** | **-0.18**  **(-0.26,**  **-0.10)** |
