# Supplementary material for: Differences in health literacy profiles of patients admitted to a public and a private hospital in Melbourne, Australia
Source: BMC Health Serv Res. 2018 Feb 22;18:134. doi: 10.1186/s12913-018-2921-4 (PMC5824469; doi:10.1186/s12913-018-2921-4)
Supplement: Supplementary file 2 — Table S2. Association between mean (SD) Health Literacy Questionnaire domain scores and health related behaviour using effect sizes, by hospital of attendance. (DOCX 49 kb) [file 12913_2018_2921_MOESM2_ESM.docx]

Additional file 2: Table S2 Association between mean (SD) Health Literacy Questionnaire domain scores and health related behaviour using effect sizes, by hospital of attendance

|  | | **Healthcare provider support**^#^ | | **Having sufficient information**^#^ | | **Actively managing health**^#^ | | **Social support for health**^#^ | | **Active appraisal of health information**^#^ | | **Active engagement with healthcare**^##^ | | | **Navigating the healthcare system**^##^ | | | **Ability to find good health information**^##^ | | | **Understanding health information**^##^ | | |
| --- | --- | --- | --- | --- | --- | --- | --- | --- | --- | --- | --- | --- | --- | --- | --- | --- | --- | --- | --- | --- | --- | --- | --- |
|  |  |  |  |  |  |  |  |  |  |  |  |  |  |  |  |  |  |  |  |  |  |  |  |
|  |  | Public | Private | Public | Private | Public | Private | Public | Private | Public | Private | Public | Private | Public | | Private | Public | | Private | Public | | Private |  |
| Sex | Female (Public n=187, Private n=1604) | 3.13  *(0.58)* | 3.34  *(0.48)* | 2.94  *(0.51)* | **3.12**  ***(0.48)*** | 2.88  *(0.53)* | **3.12**  ***(0.48)*** | 3.06  *(0.55)* | 3.25  *(0.50)* | 2.84  *(0.53)* | **2.92**  ***(0.53)*** | 3.82  *(0.74)* | 4.05  *(0.60)* | 3.62  *(0.75)* | | 3.91  *(0.58)* | 3.59  *(0.77)* | | **3.90**  ***(0.63)*** | 3.88  *(0.76)* | | 4.15  *(0.58)* |  |
|  | Male (Public n=195, Private n=1487) | 3.12  *(0.55)* | 3.35  *(0.48)* | 2.99  *(0.52)* | **3.02**  ***(0.47)*** | 2.98  *(0.51)* | **3.03**  ***(0.49)*** | 3.16  *(0.54)* | 3.26  *(0.48)* | 2.80  *(0.51)* | **2.77**  ***(0.52)*** | 3.82  *(0.82)* | 4.09  *(0.55)* | 3.65  *(0.75)* | | 3.90  *(0.55)* | 3.53  *(0.82)* | | **3.79**  ***(0.61)*** | 3.82  *(0.80)* | | 4.07  *(0.57)* |  |
|  | Effect size (95% CI) | 0.00  (-0.20,  0.21) | -0.02  (-0.09,  0.05) | -0.09  (-0.29,  0.12) | **0.22***  **(0.15,**  **0.29)** | -0.18  (-0.38,  0.02) | **0.18**  **(0.11,**  **0.25**) | -0.18  (-0.38,  0.02) | -0.03  (-0.10,  0.04) | 0.07  (-0.13,  0.27) | **0.28***  **(0.21,**  **0.35)** | -0.01  (-0.21,  0.19) | -0.09  (-0.16,  -0.02) | -0.03  (-0.23,  0.17) | | 0.02  -0.05,  0.09) | 0.08  (-0.12,  0.28) | | **0.19**  **(0.12,**  **0.26)** | 0.08  (-0.12,  0.28) | | 0.15  (0.08,  0.22) |  |
| Aged >=65 | <65 (Public n=166,  Private n=1216) | **3.06**  ***(0.62)*** | 3.33  *(0.50)* | 2.91  *(0.55)* | 3.10  *(0.48)* | 2.87  *(0.53)* | 3.09  *(0.51)* | **3.01**  ***(0.62)*** | 3.24  *(0.48)* | 2.84  *(0.48)* | 2.90  *(0.52)* | 3.77  *(0.79)* | 4.05  *(0.57)* | 3.59  *(0.75)* | | 3.89  *(0.56)* | **3.66**  ***(0.73)*** | | **3.95**  ***(0.57)*** | 3.93  *(0.69)* | | **4.17**  ***(0.54)*** |  |
|  | >=65 (Public n=216, Private n=1853) | **3.18 (*0.51)*** | 3.36  *(0.47)* | 3.01  *(0.48)* | 3.06  *(0.48)* | 2.98  *(0.51)* | 3.07  *(0.48)* | **3.18**  ***(0.46)*** | 3.27  *(0.49)* | 2.80  *(0.55)* | 2.81  *(0.53)* | 3.86  *(0.77)* | 4.08  *(0.58)* | 3.67  *(0.75)* | | 3.93  *(0.56)* | **3.48**  ***(0.84)*** | | **3.78**  ***(0.64)*** | 3.78  *(0.84)* | | **4.08**  ***(0.60)*** |  |
|  | Effect size (95% CI) | **-0.22***  **(-0.42,**  **-0.01)** | -0.06  (-0.13,  0.01) | -0.20  (-0.40,  0.01) | 0.10  (0.03,  0.17) | -0.20  (-0.40,  0.01) | 0.05  (-0.02,  0.12) | **-0.33***  **(-0.53,**  **-0.12)** | -0.06  (-0.13,  0.01) | 0.08  (-0.12,  0.29) | 0.16  (0.09,  0.23) | -0.11  (-0.31,  0.10) | -0.05  (-0.12,  0.02) | -0.10  (-0.30,  0.11) | | -0.07  (-0.14,  0.00) | **0.22***  **(0.01,**  **0.42)** | | **0.28***  **(0.21,**  **0.36)** | 0.20  (-0.01,  0.40) | | **0.16**  **(0.09,**  **0.23)** |  |
| Receives govern-ment benefits | Yes (Public n=233, Private n=1722) | 3.16  *(0.49)* | 3.37  *(0.46)* | 2.96  *0.51)* | 3.07  *(0.47)* | 2.97  *(0.49)* | 3.07  *(0.48)* | 3.13  *(0.50)* | 3.27  *(0.49)* | 2.78  *(0.53)* | 2.82  *(0.53)* | 3.81  *(0.77)* | 4.07  *(0.59)* | 3.62  *(0.77)* | | 3.91  *(0.57)* | **3.46**  ***(0.84)*** | | **3.77**  ***(0.65)*** | **3.77**  ***(0.82)*** | | **4.06**  ***(0.61)*** |  |
|  | No (Public n=133,  Private n=1325) | 3.08  *(0.63)* | 3.33  *(0.49)* | 2.97  *(0.52)* | 3.09  *(0.48)* | 2.88  *(0.53)* | 3.08  *(0.51)* | 3.08  *(0.59)* | 3.24  *(0.47)* | 2.88  *(0.47)* | 2.88  *(0.53)* | 3.84  *(0.77)* | 4.07  *(0.56)* | 3.64  *(0.72)* | | 3.90  *(0.56)* | **3.72**  ***(0.68)*** | | **3.94**  ***(0.57)*** | **3.97**  ***(0.66)*** | | **4.17**  ***(0.52)*** |  |
|  | Effect size (95% CI) | 0.14  (-0.07,  0.36) | 0.08  (0.01,  0.15) | -0.01  (-0.22,  0.21) | -0.04  (-0.11,  0.03) | 0.18  (-0.03,  0.39) | -0.02  (-0.09,  0.06) | 0.10  (-0.11,  0.32) | 0.06  (-0.01,  0.13) | -0.21  (-0.42,  0.01) | -0.12  (-0.20,  -0.05) | -0.04  (-0.25,  0.17) | 0.00  (-0.07,  0.07) | -0.03  (-0.24,  0.19) | | 0.01  (-0.06,  0.09) | **-0.32***  **(-0.53,**  **-0.10)** | | **-0.27***  **(-0.34,**  **-0.20)** | **-0.26***  **(-0.47,**  **-0.05)** | | **-0.19**  **(-0.26,**  **-0.11)** |  |
| BMI greater than 25 | Yes (Public 231 Private 1582) | 3.14  *(0.54)* | 3.36  *(0.47)* | 2.97  *(0.51)* | 3.07  *(0.47)* | 2.91  *(0.52)* | **3.04**  ***(0.49*** | 3.10  *(0.53)* | 3.25  *(0.49)* | 2.82  *(0.51)* | 2.86  *(0.53)* | 3.87  *(0.74)* | 4.09  *(0.56)* | 3.67  *(0.73)* | | 3.92  *(0.56)* | 3.61  *(0.77)* | | 3.87  *(0.61)* | 3.88  *(0.77)* | | 4.12  *(0.56)* |  |
|  | No (Public n=92,  Private n=1178) | 3.08  *(0.60)* | 3.34  *(0.49)* | 2.95  *(0.49)* | 3.09  *(0.48)* | 2.98  *(0.46)* | **3.13**  ***(0.50*** | 3.11  *(0.54)* | 3.28  *(0.47)* | 2.78  *(0.50)* | 2.84  *(0.53)* | 3.70  *(0.76)* | 4.06  *(0.59)* | 3.52  *(0.73)* | | 3.89  *(0.56)* | 3.48  *(0.79)* | | 3.85  *(0.62)* | 3.80  *(0.70)* | | 4.13  *(0.58)* |  |
|  | Effect size (95% CI) | 0.11  (-0.13,  0.35) | 0.05  (-0.02,  0.13) | 0.04  (-0.20,  0.28) | -0.04  (-0.12,  0.04) | -0.14  (-0.39,  0.10) | **-0.19**  **(-0.27,**  **-0.12)** | -0.03  (-0.27,  0.21) | -0.06  (-0.14,  0.01) | 0.09  (-0.15,  0.33) | 0.04  (-0.04,  0.12) | 0.24  (0.00,  0.48) | 0.06  (-0.01,  0.14) | 0.21  (-0.03,  0.45) | | 0.06  (-0.01,  0.14) | 0.17  (-0.08,  0.41) | | 0.03  (-0.05,  0.10) | 0.10  (-0.14,  0.34) | | -0.03  (-0.10,  0.05) |  |
| Alcohol intake greater than 2 glasses per day | Yes  (Public n=16,  Private n=214) | 3.14  *(0.55)* | 3.38  *(0.47)* | 2.92  *(0.53)* | 3.05  *(0.45)* | 2.79  *(0.61)* | **2.95**  ***(0.48)*** | 3.13  *(0.79)* | 3.21  *(0.49)* | **2.45**  ***(0.45)*** | 2.71  *(0.53)* | 3.83  *(0.83)* | 4.10  *(0.49)* | 3.57  *(0.69)* | | 3.91  *(0.51)* | 3.41  *(0.71)* | | **3.73**  ***(0.60)*** | 3.75  *(0.76)* | | 4.09  *(0.53)* |  |
|  | No (Public n=354, Private n=2823) | 3.13  *(0.55)* | 3.35  *(0.48)* | 2.97  *(0.51)* | 3.08  *(0.48)* | 2.94  *(0.51)* | **3.08**  ***(0.49)*** | 3.10  *(0.53)* | 3.26  *(0.49)* | **2.83**  ***(0.51)*** | 2.85  *(0.53)* | 3.83  *(0.77)* | 4.07  *(0.58)* | 3.64  *(0.75)* | | 3.91  *(0.57)* | 3.57  *(0.79)* | | **3.86**  ***(0.62)*** | 3.86  *(0.78)* | | 4.11  *(0.58)* |  |
|  | Effect size (95% CI) | 0.02  (-0.48,  0.52) | 0.07  (-0.07,  0.21) | -0.09  (-0.59,  0.41) | -0.05  (-0.19,  0.09) | -0.29  (-0.79,  0.21) | **-0.28***  **(-0.42,**  **-0.14)** | 0.04  (-0.46,  0.54) | -0.10  (-0.24,  0.04) | **-0.76****  **(-1.26,**  **-0.25)** | -0.28  (-0.42,  -0.14) | 0.00  (-0.50,  0.50) | 0.06  (-0.08,  0.20) | -0.09  (-0.59,  0.41) | | 0.00  (-0.14,  0.14) | -0.19  (-0.70,  0.31) | | **-0.20***  **(-0.35,**  **-0.06)** | -0.14  (-0.64,  0.36) | | -0.05  (-0.19,  0.09) |  |
| Smoking | Yes (Public n=29, Private n=96) | 3.17  *(0.69)* | **3.25**  ***(0.55)*** | 2.88  *(0.71)* | 3.00  *(0.52)* | **2.80***  ***(0.70)*** | **2.89***  ***(0.54)*** | **2.91**  ***(0.78*** | **3.12**  ***(0.63)*** | 2.77  *(0.75)* | 2.75  *(0.52* | 3.74  *(0.97)* | 3.98  *(0.69)* | 3.52  *(0.86)* | | 3.82  *(0.63)* | 3.44  *(0.90)* | | **3.68**  ***(0.73)*** | 3.73  *(0.90)* | | 4.06  *(0.57)* |  |
|  | No Public n=342,  Private =2941) | 3.12  *(0.55)* | **3.35**  ***(0.48)*** | 2.97  *(0.50)* | 3.08  *(0.47)* | **2.94***  ***(0.50)*** | **3.08***  ***(0.49)*** | **3.12**  ***(0.52)*** | **3.26**  ***(0.48)*** | 2.82  *(0.49)* | 2.85  *(0.53)* | 3.82  *(0.77)* | 4.07  *(0.57)* | 3.64  *(0.74)* | | 3.91  *(0.56)* | 3.56  *(0.79)* | | **3.85**  ***(0.62)*** | 3.85  *(0.78)* | | 4.11  *(0.58)* |  |
|  | Effect size (95% CI) | 0.09  (-0.29,  0.47) | **-0.20**  **(-0.40,**  **0.00)** | -0.18  (-0.57,  0.20) | -0.16  (-0.37,  0.04) | **-0.27***  **(-0.65,**  **0.11)** | **-0.39***  **(-0.59,**  **-0.18**) | **-0.38**  **(-0.76,**  **0.00)** | **-0.29**  **(-0.50,**  **-0.09)** | -0.09  (-0.47,  0.29) | -0.18  (-0.39,  0.02) | -0.11  (-0.49,  0.27) | -0.17  (-0.38,  0.03) | -0.17  (-0.55,  0.21) | | -0.17  (-0.37,  0.04) | -0.15  (-0.53,  0.23) | | **-0.28***  **(-0.48,**  **-0.07)** | -0.15  (-0.53,  0.23) | | -0.09  (-0.29,  0.12) |  |
| Physical Activity >=2.5 hrs/ week | Yes (Public n=211, Private n=535) | 3.13  *(0.58)* | 3.36  *(0.49)* | 2.98  *(0.52)* | 3.16  *(0.48* | **2.99**  ***(0.51)*** | **3.27**  ***(0.48)*** | 3.14  *(0.53)* | 3.27  *(0.48)* | 2.83  *(0.52)* | **2.92**  ***(0.53)*** | 3.85  *(0.72)* | 4.07  *(0.57)* | 3.67  *(0.68)* | | 3.90  *(0.57)* | 3.60  *(0.73)* | | 3.94  *(0.61)* | **3.92**  ***(0.69)*** | | 4.16  *(0.58)* |  |
|  | No (Public n=172, Private n=2557) | 3.11  *(0.55)* | 3.34  *(0.48)* | 2.95  *(0.51)* | 3.05  *(0.48)* | **2.85**  ***(0.53)*** | **3.03**  ***(0.48)*** | 3.06  *(0.57)* | 3.25  *(0.49)* | 2.80  *(0.52)* | **2.83**  ***(0.53)*** | 3.79  *(0.84)* | 4.07  *(0.58)* | 3.59  *(0.83)* | | 3.91  *(0.56)* | 3.51  *(0.87)* | | 3.83  *(0.62)* | **3.76**  ***(0.88)*** | | 4.10  *(0.57)* |  |
|  | Effect size (95% CI) | 0.03  (-0.17,  0.24) | 0.03  (-0.06,  0.12) | 0.06  (-0.14,  0.26) | 0.22  (0.12,  0.31) | **0.27***  **(0.07,**  **0.47)** | **0.50****  **(0.41,**  **0.59)** | 0.15  (-0.05,  0.35) | 0.04  (-0.05,  0.13) | 0.06  (-0.14,  0.26) | **0.17**  **(0.08,**  **0.26)** | 0.08  (-0.12,  0.28) | 0.00  (-0.09,  0.09) | 0.10  (-0.11,  0.30) | | -0.02  (-0.11,  0.08) | 0.11  (-0.09,  0.32) | | 0.18  (0.09,  0.27) | **0.21***  **(0.00,**  **0.41)** | | 0.10  (0.01,  0.19) |  |

^#^Scale range 0-4, higher score indicates greater ability or more support; ^##^Scale range 0-5, higher score indicates greater ability or more support. Variables in **bold** are significant at the 0.05 level
